# OTUD3 suppresses the mTORC1 signaling by deubiquitinating KPTN

**DOI:** 10.3389/fphar.2023.1337732

**Published:** 2024-01-15

**Authors:** Jiatao Li, Dan Yang, Yan Lin, Wei Xu, Shi-min Zhao, Chenji Wang

**Affiliations:** ^1^ Institutes of Biomedical Sciences, Obstetrics & Gynecology Hospital of Fudan University, Institutes of Metabolism and Integrative Biology, State Key Laboratory of Genetic Engineering, MOE Engineering Research Center of Gene Technology, School of Life Sciences, Fudan University, Shanghai, China; ^2^ Department of Orthopedics, Shanghai Children’s Hospital, School of Medicine, Shanghai Jiao Tong University, Shanghai, China

**Keywords:** OTU domain-containing protein 3, KPTN, deubiquitination, mTORC1, KICSTOR

## Abstract

**Background:** Ubiquitination and deubiquitination modifications play pivotal roles in eukaryotic life processes, regulating protein dynamics via the ubiquitin-proteasome pathway. Dysregulation can impact disease development, including cancer and neurodegenerative disorders. Increasing evidence highlights their role in tumorigenesis, modulating key proteins. OTUD3, a deubiquitinase, stabilizes PTEN, suppressing tumor growth by inhibiting PI3K-AKT signaling. Yet, further OTUD3 substrates remain underexplored.

**Methods:** We employed the *In vivo* ubiquitination assay to investigate the ubiquitination role of OTUD3 on KPTN within the cellular context. Additionally, CRISPR/Cas9 editing and Immunofluorescence were utilized to study the impact of OTUD3 on the mTOR signaling pathway in cells. Furthermore, Cell proliferation assay and NMR were employed to explore the effects of OTUD3 on cellular growth and proliferation.

**Results:** OTUD3 serves as a deubiquitinase for KPTN. OTUD3 interacts with KPTN, facilitated by the OTU domain within OTUD3. Further investigations confirmed KPTN’s ubiquitination modification, primarily at lysine residue 49. Ubiquitination experiments demonstrated OTUD3’s ability to mediate KPTN’s deubiquitination without affecting its protein levels. This suggests KPTN’s ubiquitination is a function-regulated, non-degradable modification. Under various amino acid starvation or stimulation conditions, overexpressing OTUD3 reduces mTORC1 signaling activation, while knocking out OTUD3 further enhances it. Notably, OTUD3’s regulation of mTORC1 signaling relies on its deubiquitinase activity, and this effect is observed even in PTEN KO cells, confirming its independence from PTEN, a reported substrate. OTUD3 also promotes GATOR1’s lysosomal localization, a process requiring KPTN’s involvement. Ultimately, OTUD3 affects cellular metabolic pool products by downregulating the mTORC1 pathway, significantly inhibiting tumor cell growth and proliferation.

**Discussion:** Our experiments shed light on an alternative perspective regarding the intrinsic functions of OTUD3 in inhibiting tumor development. We propose a novel mechanism involving KPTN-mediated regulation of the mTORC1 signaling pathway, offering fresh insights into the occurrence and progression of tumor diseases driven by related genes. This may inspire new approaches for drug screening and cancer treatment, potentially guiding future therapies for relevant tumors.

## 1 Introduction

Ubiquitination/deubiquitination modification participates in almost all life activities of eukaryotes, and the ubiquitin-proteasome degradation pathway is an important mechanism for regulating protein expression level, stability and activity (Li et al., 2022; Yang et al., 2023). Abnormal changes in the ubiquitination process will affect the occurrence and development of a series of diseases, such as cancer, diabetes, Parkinson’s disease and other neurodegenerative diseases. More and more evidences show that ubiquitination/deubiquitination modification affects the occurrence and development of tumors by regulating the stability and activity of important oncoproteins or tumor suppressor proteins (Jiang et al., 2022; Zhang et al., 2023a). It has been reported that the deubiquitinating enzyme family OTUD3 can directly interact with the tumor suppressor protein PTEN and stabilize PTEN through deubiquitination modification, thereby antagonizing the PI3K-AKT signaling pathway and exerting a tumor suppressor function (Yuan et al., 2015; ZhEl-Kott et al., 2020). However, little is known about the existence of other deubiquitination substrates for OTUD3. In order to identify other important biological functions of the deubiquitinating enzyme OTUD3, we used protein affinity purification and mass spectrometry methods to find new OTUD3 interacting proteins. Through this method, we found that there is a potential interaction between the deubiquitinating enzyme OTUD3 and KPTN, which is one of the components of the KICSTOR protein complex, an important regulatory factor in the mTORC1 signaling pathway.

Researchers have identified gene mutations in the breast cancer cell lines involving OTUD3, leading to loss of protein function and accelerating the migration and metastasis of cancer cells. Subsequently, the most notable function of OTUD3 was discovered by researchers, as it relates to the deubiquitination (DUB) of the tumor suppressor PTEN, ensuring its stability. PTEN is one of the most commonly mutated tumor suppressors, and the reduction in PTEN protein stability also plays a role in tumorigenesis. OTUD3 interacts with PTEN, removing its polyubiquitination and promoting its stability. Depletion of OTUD3 results in the activation of Akt signaling, inducing cellular transformation and facilitating cancer metastasis (Yuan et al., 2015). Furthermore, researchers have observed in various databases that cancer patients with tumors of different locations and types exhibit one or more mutation sites with amino acid substitutions in the OTUD3 gene. These mutation sites are situated within or immediately following the OTU domain or its adjacent amino acid residues.

mTOR is an atypical serine/threonine protein kinase that resides within two distinct protein complexes. Much like the promising effects demonstrated by rapamycin in clinical treatment, mTOR also occupies a central position in numerous major signaling pathways, orchestrating the normal physiological processes of cells and tissues (Laplante and Sabatini, 2012). mTOR integrates intracellular energy, nutrient signals, as well as extracellular environmental factors such as hormone levels, growth factors, and oxygen signals, to coordinate cellular anabolic and catabolic metabolism. Dysregulation of the mTOR signaling pathway disrupts cellular homeostasis, leading to severe consequences such as malignant transformation, tumor formation, autophagy, and ultimately human diseases (Liu et al., 2022; Zhan et al., 2023).

Typically, mTORC1 remains relatively inactive until it translocates to the catalytic site upon binding to a small GTPase called Rheb, which becomes activated (Yang et al., 2017). The majority of upstream input signals in the mTOR pathway are channeled through PI3K/Akt and TSC1/2 to activate Rheb. Amino acids, on the other hand, transmit signals independently through distinct sensors to the complex composed of RagA/B and RagC/D, situated upstream of mTORC1. Both mechanisms ultimately lead to the recruitment and activation of mTORC1 on the membrane surface of lysosomes by activated Rheb. The activation process of mTORC1 is also directly or indirectly regulated by a series of protein complexes, including Ragulator (Bar-Peled et al., 2012), Rag GTPases (Sancak et al., 2008), GATOR1/2 (Bar-Peled et al., 2013), KICSTOR (Wolfson et al., 2017), and others.

## 2 Materials and methods

### 2.1 Cell lines and cell culture

HEK293T (ATCC Number: CRL-11268), HeLa (ATCC Number: CCL-2) were purchased from Shanghai Cell Bank and tested negative for *mycoplasma* contamination. HeLa cells were authenticated using Short Tandem Repeat (STR) analysis by Shanghai Biowing Applied Biotechnology Company. HeLa and HEK293T cells were cultured in DMEM (HyClone) supplemented with 10% newborn bovine serum (HyClone), 100 units mL^−1^ penicillin, and 100 μg mL^−1^ streptomycin (Invitrogen). For ubiquitination assays, the proteasome inhibitor, MG132, was added 4 h before harvesting the cells.

### 2.2 Plasmids and antibodies

Polymerase chain reaction (PCR)-amplified CDS of human OTUD3 and its variants, KPTN, ITFG2, SZT2 was cloned into pCMV vector between EcoRI and NotI.

The antibody against for OTUD3 (#ab107646, dilution 1:100), KPTN (#ab32199, Y184, dilution 1:500) was purchased from ABcam. The anti-Flag mouse mAb (3Bg) (#M20008, dilution 1:3000), anti-Myc mouse mAb (19C2) (#M20002, dilution 1:3000), anti-HA mouse mAb (26D11) (#M20003, dilution 1:3000) antibodies were obtained from Abmart. The p70 S6 kinase (#9202, dilution 1:1000), phospho-p70 S6 kinase (Thr389) (#9205, dilution 1:1000) antibodies were obtained from Cell Signaling Technology.

### 2.3 Cell transfections, immunoprecipitation, and immunoblotting

Plasmid transfections were carried out by the Polyethylenimine (PEI), Lipofectamine 2000 (Invitrogen), or calcium phosphate methods. In the PEI transfection method, 500 μL of DMEM (serum-free medium) and the plasmid were placed in an empty EP tube and PEI (three times the concentration of plasmid) was added into the medium with vigorous shaking. The mixture was incubated for 15 min. Meanwhile, the cell culture medium was replaced with 2 mL of fresh 10% NCS medium. After 15 min, the mixture was added to the cells, and the fresh medium was replaced after 12 h. After 36 h, the transfection was completed and the cells were treated or harvested. In the Lipofectamine 2000 transfection method. DMEM (250 μL) was added to two clean EP tubes and 6 μL of Lipofectamine 2000 was added to one of the tubes and mixed for 5 min The plasmid was added in the other tube and then added to the medium containing Lipofectamine 2000, mixed, and allowed to stand for 20 min Meanwhile, the cell culture medium was replaced by serum-free medium as serum interferes with Lipofectamine 2000 transfection efficiency. Six hours after the addition of the mixture to the cells, the medium was replaced with fresh normal medium and cells were collected 36 h later. In the calcium phosphate method, we aspirated the medium and added 9 mL fresh DMEM first, and then placed the cells back into the incubator for at least 1 h. This is important to balance the pH for transfection efficiency. The DNA in ddH2O (up to 450 μL) was mixed with 500 μL of 2 × HBS buffer and 50 μL of CaCl2 was added drop by drop with shaking. The mixture was incubated on ice for 10 min Chloroquine (2000×, 5 μL) was added to the cells and the mixture was added drop by drop into the plates gently. The plates were swirled and placed back into the incubator. After 5–6 h after transfection, the medium was aspirated and the cells were washed twice with PBS and fresh medium was added. The cells were collected 24–48 h later.

For immunoprecipitation, cells were lysed with 0.5% NP-40 buffer containing 50 mM Tris-HCl (pH 7.5), 150 mM NaCl, 0.3% NONIDET P-40, 1 μg mL^−1^ aprotinin, 1 μg mL^−1^ leupeptin, 1 μg mL^−1^ pepstatin, and 1 mM PMSF. Cell lysates were incubated with Flag beads (Sigma) for 3 h at 4 °C. The binding complexes were washed with 0.5% NP-40 buffer and mixed with loading buffer for SDS-PAGE.

### 2.4 *In vivo* ubiquitination assay

HA-tagged ubiquitin and target plasmids were transfected for 36 h. MG132 was added for 4 h before harvesting the cells to inhibit proteasome activity. Cells were next lysed in 1% SDS buffer (Tris-HCl pH 7.5, 0.5 mM EDTA, 1 mM DTT), and boiled for 10 min When the cell pellets became clear, the lysates were diluted 10-fold in Tris-HCl Buffer. Flag Beads (Sigma) was used for immunoprecipitation. Ubiquitination was analyzed by immunoblotting using anti-HA and anti-Flag antibodies.

### 2.5 Gene knocked out cell line generation

To generate OTUD3-knockout HeLa cells, we used the following guide sequence targeting the human OTUD3, 5′-GAG​GTG​ACC​TAC​CTG​CCA​CA-3′. To generate PTEN-knockout HeLa cells, we used the following guide sequence targeting the human PTEN, 5′-GAA​ATT​CGG​GCT​ATT​CTG​CA-3′. To generate KPTN-knockout HeLa cells, we used the following guide sequence targeting the human KPTN, 5′-gtg​aag​ctg​tcc​tcg​cgc​aa-3′.

### 2.6 Cell proliferation assay

Cell proliferation was assessed using the CCK-8 assay kit, following the specific steps outlined below: Cells were detached using trypsin, and then seeded onto a 96-well plate at a density of 4 × 103 cells per well. Each treatment was performed in quadruplicate, and the cells were allowed to adhere for 12 h to ensure proper attachment. Following various treatments, 10 μL of CCK-8 reagent was added to each well, including four control wells with only culture medium and CCK-8, but no cells. The plate was then incubated at 37°C for 2 h. The absorbance at 450 nm (O.D.450 nm) was measured using a microplate reader, and the values obtained represented the absorbance on the first day. The same procedure was repeated daily at the same time over a period of 5 days. The O.D. values were corrected by subtracting the blank control, and the resulting values were plotted on a graph with time on the x-axis and O.D. values on the y-axis to generate proliferation curves for cells under different treatments.

### 2.7 Immunofluorescence and confocal microscopy

The cells were seeded on glass coverslips in 24-well plates and harvested at 80% confluence, and were washed with PBS and fixed with 4% paraformaldehyde in PBS. After permeabilization with 0.4% Triton X-100 for 10 min and then in the blocking solution (PBS plus 5% donkey serum), for 30 min at room temperature (RT). The cells were then incubated with primary antibodies at 4°C overnight. After washing with PBST buffer, fluorescence-labelled secondary antibodies were applied. DAPI was utilized to stain nuclei. The glass coverslips were mounted on slides and imaged using a confocal microscope (LSM880, Zeiss) with a 63*/1.4NA Oil PSF Objective. Quantitative analyses were performed using ImageJ software.

### 2.8 Statistical analysis

Graph plotting and statistical assessments were executed utilizing GraphPad Prism (version 9). Microscopy images were randomly selected from diverse regions. Pertinent information including sample size (n), employed statistical test, and corresponding *p*-value for each experiment were provided within the figure captions. The findings are depicted as median ±SD or SEM. Statistical distinctions between the two groups were evaluated using Student’s t-test. In scenarios involving multiple treatment groups and a single control group, a one-way analysis of variance (ANOVA) was employed. Statistical significance was recognized for *p*-values < 0.05.

## 3 Results

### 3.1 OTU-domain of OTUD3 interacted with KPTN and KICSTOR

**FIGURE 1:**
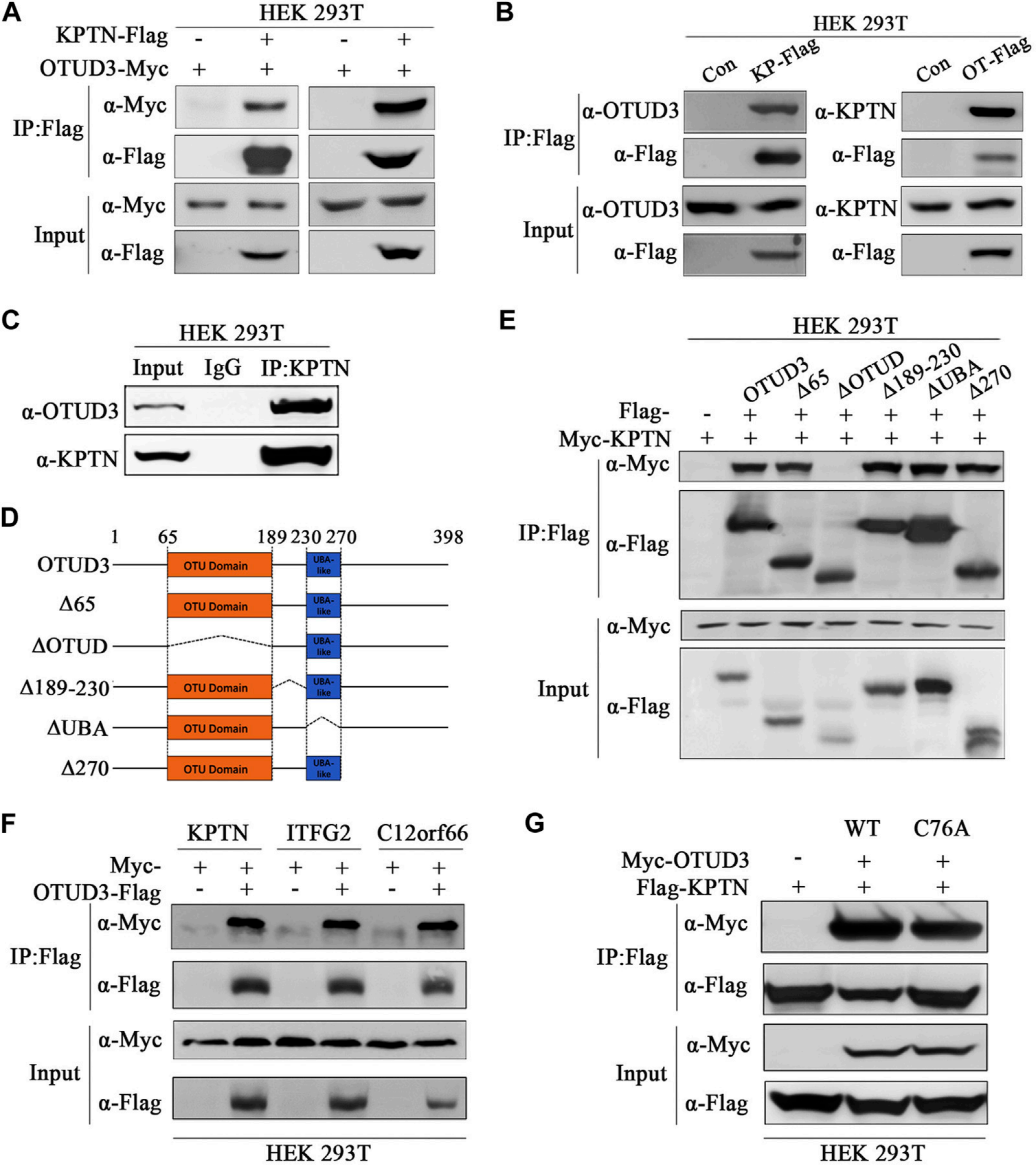
OTU-domain of OTUD3 interacted with KPTN and KICSTOR. **(A)** In HEK-293T cells, exogenous expression of OTUD3-Flag and KPTN-Myc was conducted, and immunoprecipitation of OTUD3-Flag was enriched using anti-Flag magnetic beads. The results demonstrate successful immunoprecipitation of OTUD3-Flag. “Input” refers to the cell lysate fraction before magnetic bead enrichment, and “IP” refers to the protein samples after washing and elution of the immunoprecipitates. **(B)** In HEK-293T cells, Flag-tagged KPTN or OTUD3 was individually expressed, and exogenous proteins were immunoprecipitated using anti-Flag magnetic beads. Co-immunoprecipitated protein components were detected using corresponding endogenous antibodies. In HEK-293T cells, Flag and OTUD3-Myc were co-expressed, and KPTN-Flag was immunoprecipitated using anti-Flag magnetic beads. Co-immunoprecipitated OTUD3-Myc was detected using anti-Myc antibodies. “Input” refers to the cell lysate fraction before magnetic bead enrichment, and “IP” refers to the protein samples after washing and elution of the immunoprecipitates. **(C)** Endogenous immunoprecipitation experiment using KTPN’s endogenous antibodies and IgG. They were used to bind endogenous OTUD3 proteins in HEK293T cell lysates. Then, protein A-coupled magnetic beads were used to enrich endogenous antibodies. The collected proteins were detected using antibodies corresponding to OTUD3 and KPTN. **(D)** Comparative schematic of various OTUD3 truncation mutants with the wild type. Dotted lines represent the truncated regions. Numbers represent the amino acid positions. The OTU domain of the OTUD3 protein starts at the 65th amino acid and ends at the 189th amino acid, while the UBA-like domain starts at the 230th amino acid and ends at the 270th amino acid. **(E)** In HEK-293T cells, OTUD3-Flag, truncated OTUD3-Δ65-Flag, OTUD3-ΔOTUD-Flag, OTUD3-Δ189-230-Flag, OTUD3-ΔUBA-Flag, OTUD3-Δ270-Flag, and KPTN-Myc were exogenously expressed. KPTN-Flag was immunoprecipitated using anti-Flag magnetic beads, and co-immunoprecipitated protein components were detected using anti-Myc antibodies. “Input” refers to the cell lysate fraction before magnetic bead enrichment, and “IP” refers to the protein samples after washing and elution of the immunoprecipitates. **(F)** In HEK-293T cells, KPTN-Myc, ITFG2-Myc, C12orf66-Myc, and OTUD3-Flag were exogenously expressed. OTUD3-Flag was immunoprecipitated using anti-Flag magnetic beads, and co-immunoprecipitated protein components were detected using anti-Myc antibodies. It was found that OTUD3 interacts with all of them. **(G)** In HEK-293T cells, OTUD3-Myc, OTUD3C76A-Myc, and KPTN-Flag were exogenously expressed. KPTN-Flag was immunoprecipitated using anti-Flag magnetic beads, and co-immunoprecipitated protein components were detected using anti-Myc antibodies. “Input” refers to the cell lysate fraction before magnetic bead enrichment, and “IP” refers to the protein samples after washing and elution of the immunoprecipitates.

In our preliminary mass spectrometry results, we found an interaction between the deubiquitinase OTUD3 and a component of the mTORC1 signaling pathway, KPTN. To confirm this interaction, we overexpressed OTUD3-Myc and KPTN-Flag in HEK-293T cells and performed immunoprecipitation experiments, which showed a clear interaction between the two proteins (Figure 1A). Additionally, we validated this interaction by reciprocal experiments, where we overexpressed KPTN-Myc and OTUD3-Flag and obtained similar results (Figure 1A). Further confirmation involved separate overexpression of KPTN-Flag and OTUD3-Flag in HEK-293T cells, showing their interaction with endogenous KPTN and OTUD3, respectively (Figure 1B). We performed co-immunoprecipitation using endogenous KPTN antibody, reinforcing their interaction (Figure 1C). Then we investigated the crucial domains of OTUD3 responsible for its interaction with KPTN. OTUD3 consists of two main domains: the OTU domain (amino acids 65–189) and the UBA-like domain (amino acids 230–270).

To determine which domain is pivotal for the interaction, we generated truncated OTUD3 constructs. Our experiments indicated that the OTU domain plays a pivotal role in the interaction between OTUD3 and KPTN (Figures 1D, E). Considering KPTN’s involvement in the KICSTOR complex, we explored whether OTUD3 interacts with other components of KICSTOR. We overexpressed KPTN-Myc, ITFG2-Myc, and C12orf66-Myc in HEK-293T cells, and co-immunoprecipitation experiments showed interactions between OTUD3 and all three KICSTOR components (Figure 1F). Additionally, we generated a catalytically inactive mutant of OTUD3 (OTUD3C76A) for future experiments. We performed co-immunoprecipitation with KPTN-Flag using OTUD3-Myc and OTUD3C76A-Myc and found that the enzymatically inactive OTUD3C76A retains its ability to interact with KPTN-Flag (Figure 1G).

Thus, these results validated the interaction between OTUD3 and KPTN. Meanwhile, we determined that the OTU domain of OTUD3 is crucial for this interaction and found evidence of OTUD3’s interaction with other KICSTOR components as well.

### 3.2 OTUD3 promoted the deubiquitination of KPTN but did not affect its protein levels

**FIGURE 2:**
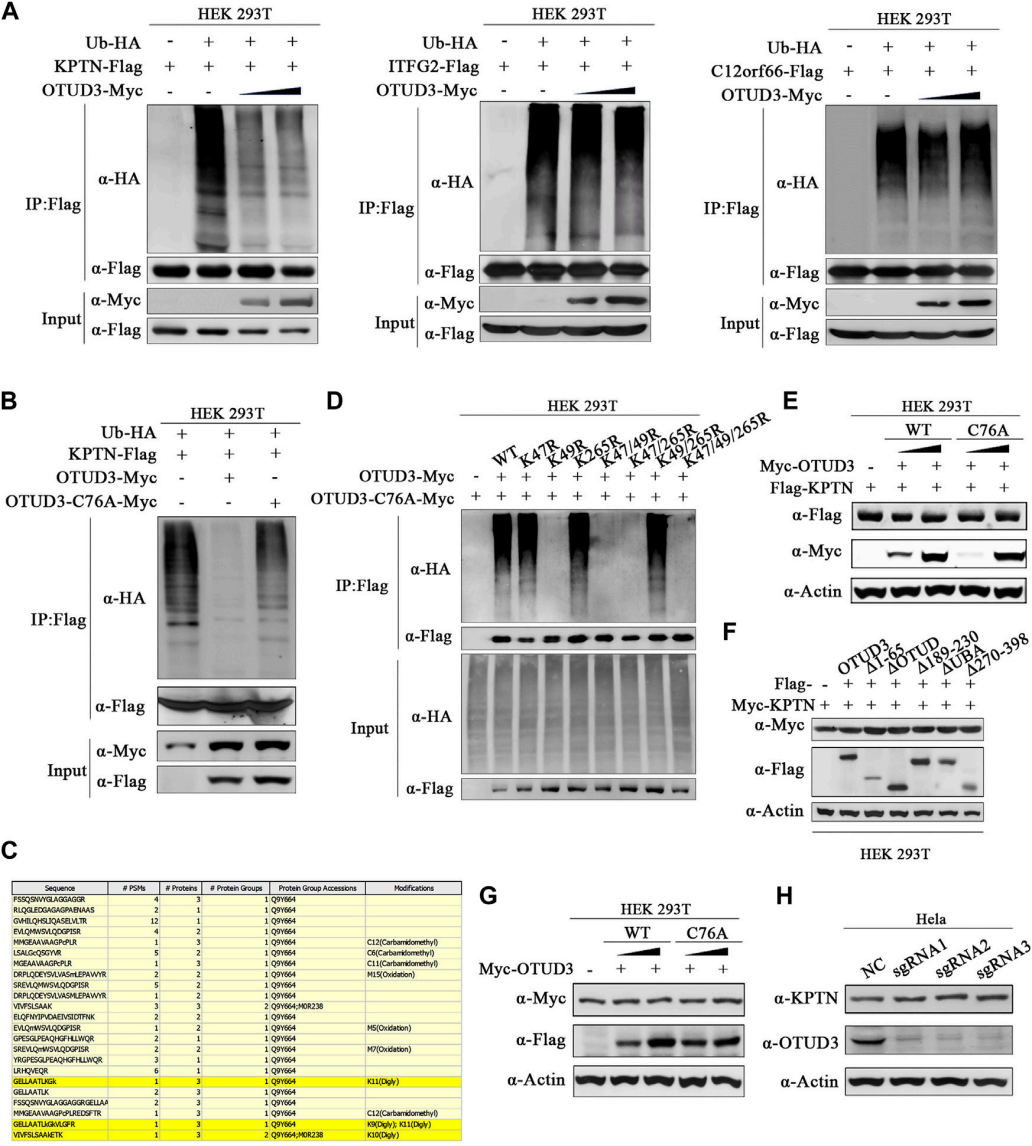
OTUD3 promoted the deubiquitination of KPTN but did not affect its protein levels. **(A)** In HEK-293T cells, exogenous expression of KPTN-Flag, ub-HA, and density gradient OTUD3-Myc was performed. KPTN-Flag ubiquitination modification decreases with increasing OTUD3, as detected using anti-HA antibodies. In HEK-293T cells, exogenous expression of ITFG2-Flag, ub-HA, and density gradient OTUD3-Myc was conducted, but there was no significant change in the ubiquitination modification of ITFG2-Flag, as detected using anti-HA antibodies. Similarly, in HEK-293T cells, exogenous expression of C12orf66-Flag, ub-HA, and density gradient OTUD3-Myc showed no significant change in the ubiquitination modification of C12orf66-Flag, as detected using anti-HA antibodies. “Input” refers to the cell lysate fraction before magnetic bead enrichment, and “IP” refers to the protein samples after washing and elution of the immunoprecipitates. **(B)** In HEK-293T cells, exogenous expression of KPTN-Flag, ub-HA, and either OTUD3-Myc or OTUD3C76A-Myc was carried out. KPTN-Flag ubiquitination modification decreases significantly when OTUD3C76A is present, as detected using anti-HA antibodies. **(C)** Yellow highlights represent peptide sequences with ubiquitination modifications. **(D)** HEK-293T cells were co-expressing Ub-HA and KPTN-Flag, KPTNK47R-Flag, KPTNK49R-Flag, KPTNK265R-Flag, KPTNK47/49R-Flag, KPTNK47/265R-Flag, KPTNK49/265R-Flag, KPTNK47/49/265R-Flag. KPTN-Flag and its various mutants were immunoprecipitated using anti-Flag magnetic beads, and the ubiquitination modification of KPTN-Flag was examined using anti-HA antibodies. Only the mutant with the K49R mutation showed a significant reduction in ubiquitination modification. **(E)** In HEK-293T cells, exogenous expression of KPTN-Flag and density gradient OTUD3-Myc or OTUD3C76A-Myc showed no significant change in the protein levels of KPTN-Flag, as detected using anti-Flag antibodies. **(F)** In HEK-293T cells, exogenous expression of OTUD3-Flag, truncated OTUD3-Δ65-Flag, OTUD3-ΔOTUD-Flag, OTUD3-Δ189-230-Flag, OTUD3-ΔUBA-Flag, OTUD3-Δ270-Flag, and KPTN-Myc showed no significant change in the protein levels of KPTN-Myc, as detected using anti-Myc antibodies. **(G)** In HEK-293T cells, exogenous expression of density gradient OTUD3-Myc or OTUD3C76A-Myc showed no significant change in the endogenous protein levels of KPTN, as detected using KPTN’s endogenous antibodies. **(H)** Protein levels of KPTN in HeLa cells with OTUD3 knockout. Using KPTN’s endogenous antibodies, there was no significant change in the endogenous protein levels of KPTN in OTUD3-knockout HeLa cells.

It remains uncertain whether all these interactions between OTUD3 and KICSTOR have biological significance and whether all the KICSTOR components substrates of OTUD3’s deubiquitination activity. So, to investigate the potential ubiquitination of KPTN and OTUD3’s ability to remove its ubiquitination, we performed the following experiments. Overexpressed KPTN-Flag and Ub-HA HEK-293T cells, along with varying levels of OTUD3-Myc or enzymatically inactive OTUD3C76A-Myc, were established. Results revealed that exogenous KPTN was indeed ubiquitinated, and the presence of OTUD3 reduced the level of KPTN ubiquitination in a manner directly proportional to OTUD3 protein levels. However, similar experiments on ITFG2 and C12orf66 showed that OTUD3 did not affect their ubiquitination levels (Figure 2A). Furthermore, a comparison between enzymatically active OTUD3 and the inactive mutant OTUD3C76A indicated that the deubiquitination ability of OTUD3C76A towards KPTN was significantly lower than that of the wild-type OTUD3 (Figure 2B).

To uncover the potential ubiquitination sites on KPTN, we performed tandem affinity purification (TAP) followed by mass spectrometry. We identified three potential ubiquitination sites on KPTN about K47, K49, and K265 (Figure 2C). Subsequent experiments involving single-point and multi-point mutants confirmed that mutating the 49th lysine residue significantly reduced KPTN ubiquitination, whereas mutations at positions 47 and 265 had minimal impact (Figure 2D). Then we examined whether OTUD3’s deubiquitination prevents KPTN degradation or affects its functionality. We found that neither wild-type OTUD3 nor the enzymatically inactive OTUD3C76A influenced exogenous KPTN protein levels (Figure 2E), even when testing various truncated forms of OTUD3 (Figure 2F). Additional experiments with endogenous KPTN further supported these findings, demonstrating that neither wild-type nor enzymatically inactive exogenous OTUD3 impacted endogenous KPTN levels (Figure 2G).

These results collectively suggest that OTUD3’s deubiquitination activity or its presence does not affect KPTN protein levels. Further confirmation came from CRISPR/Cas9-based knockout of the OTUD3 gene in HeLa cells (OTUD3-KO-HeLa), which showed that endogenous KPTN levels remained unchanged in the absence of OTUD3 (Figure 2H). These findings underscore that OTUD3’s deubiquitination activity or its presence does not alter KPTN protein levels.

### 3.3 OTUD3 downregulated mTORC1 signaling pathway on account of its enzymatic deubiquitinase activity

**FIGURE 3:**
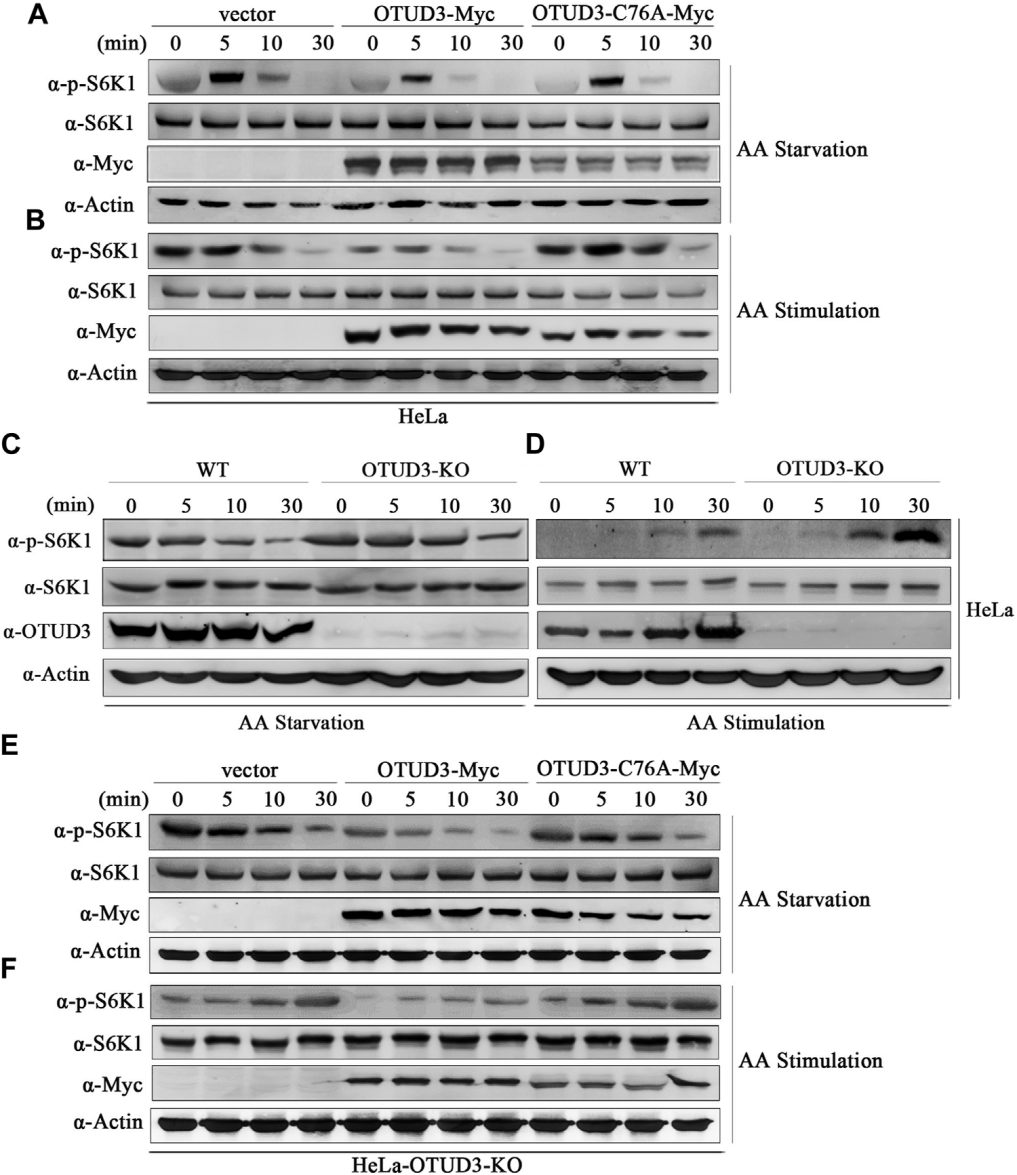
OTUD3 downregulated mTORC1 signaling pathway on account of its enzymatic deubiquitinase activity. **(A)** Wild-type HeLa cells and HeLa cells with exogenous expression of OTUD3-Myc and OTUD3C76A-Myc were subjected to a time gradient amino acid starvation treatment using amino acid-free basal culture medium. Changes in S6K1 phosphorylation were examined. **(B)** Wild-type HeLa cells and HeLa cells with exogenous expression of OTUD3-Myc and OTUD3C76A-Myc were subjected to amino acid starvation for 1 h using amino acid-free basal culture medium, followed by amino acid treatment for a specified duration. Changes in S6K1 phosphorylation were examined. **(C)** OTUD3 knockout or wild-type HeLa cells were subjected to a time gradient amino acid starvation treatment using amino acid-free basal culture medium. Changes in S6K1 phosphorylation were examined. **(D)** OTUD3 knockout or wild-type HeLa cells were subjected to amino acid starvation for 1 h using amino acid-free basal culture medium, followed by amino acid treatment for a specified duration. Changes in S6K1 phosphorylation were examined. **(E)** OTUD3 knockout HeLa cells and OTUD3 knockout HeLa cells with exogenous expression of OTUD3-Myc and OTUD3C76A-Myc were subjected to a time gradient amino acid starvation treatment using amino acid-free basal culture medium. Changes in S6K1 phosphorylation were examined. **(F)** OTUD3 knockout HeLa cells and OTUD3 knockout HeLa cells with exogenous expression of OTUD3-Myc and OTUD3C76A-Myc were subjected to amino acid starvation for 1 h using amino acid-free basal culture medium, followed by amino acid treatment for a specified duration. Changes in S6K1 phosphorylation were examined. “AA” stands for amino acid.

KPTN is part of the protein complex KICSTOR located in the mTORC1 signaling pathway, recruiting GATOR1 to the lysosomal membrane. GATOR1 deactivates RagA and RagB, reducing the formation of Rag GTPase A/B heterodimers and their interaction with mTORC1. This impedes the localization of mTORC1 to lysosomes, leading to the downregulation of the mTORC1 signaling pathway (Sancak et al., 2010). Therefore, does OTUD3-mediated deubiquitination of KPTN affect the mTORC1 signaling pathway and what is its functional significance. Thus, to explore this issue we transiently transfected wild-type HeLa cells with OTUD3-Myc and catalytically inactive OTUD3C76A-Myc, using HeLa cells transfected with an empty vector as a control. These cells were subjected to a time-gradient of amino acid starvation or amino acid stimulation after starvation. The results of immunoblot with specific endogenous antibodies indicated that in wild-type HeLa cells, the phosphorylation level of S6K1 gradually decreased with prolonged amino acid starvation and increased with prolonged amino acid stimulation (Figure 3A). Interestingly, in wild-type HeLa cells overexpressing OTUD3-Myc, although the phosphorylation trend of S6K1 was similar, the overall phosphorylation level was significantly reduced compared to wild-type HeLa cells (Figures 3A, B). These findings suggested that exogenous OTUD3 has a suppressive effect on the mTORC1 signaling pathway.

Furthermore, we found that exogenous OTUD3 expression had a downregulatory effect on the mTORC1 signaling pathway. Our study revealed that in HeLa cells expressing either wild-type OTUD3-Myc or catalytically inactive OTUD3C76A-Myc, the phosphorylation trend of S6K1 was similar. However, when compared to HeLa cells overexpressing wild-type OTUD3-Myc, HeLa cells overexpressing catalytically inactive OTUD3C76A-Myc exhibited an overall increase in S6K1 phosphorylation levels, which were closer to those of the wild-type HeLa control group (Figures 3A, B). Moreover, during amino acid starvation, HeLa cells overexpressing wild-type OTUD3-Myc showed a faster decrease in S6K1 phosphorylation levels, while during amino acid stimulation after starvation, the increase was slower. In HeLa cells overexpressing catalytically inactive OTUD3C76A-Myc, the changes in S6K1 phosphorylation levels were more gradual. These experiments to some extent suggest that the OTUD3 protein can exert a downregulatory effect on the mTORC1 signaling pathway, and this downregulation relies on the enzymatic deubiquitinase activity of OTUD3.

To further validate our hypothesis, we selected wild-type HeLa cells (WT) and OTUD3 knockout HeLa cells (OTUD3-KO) as study subjects. These cells were subjected to a time-gradient of amino acid starvation or amino acid stimulation after starvation. The results of immunoblot with specific endogenous antibodies indicated that in both WT and OTUD3-KO HeLa cells, the phosphorylation level of S6K1 decreased with prolonged amino acid starvation and increased with prolonged amino acid stimulation (Figure 3C). Although the phosphorylation trend of S6K1 was similar, the overall phosphorylation level of S6K1 in OTUD3-KO cells was higher compared to WT cells (Figures 3C, D). In the absence of OTUD3, the mTORC1 signaling pathway was upregulated, providing further evidence for the suppressive effect of OTUD3 on the mTORC1 signaling pathway.

Subsequently, we designed rescue experiments in OTUD3 knockout HeLa cells. We reintroduced exogenous OTUD3-Myc and catalytically inactive OTUD3C76A-Myc into these cells and subjected them to a time gradient of amino acid starvation or amino acid stimulation after starvation. The results of immunoblot with corresponding endogenous antibodies revealed that compared to OTUD3-KO cells, the overall phosphorylation level of S6K1 was downregulated in cells with reintroduced exogenous OTUD3 (Figures 3E, F). This result indicates that the upregulation of the mTORC1 signaling pathway caused by OTUD3-KO cells can be counteracted by exogenous expression of WT cells. Additionally, we observed that in OTUD3-KO cells, compared to the reintroduction of exogenous wild-type OTUD3, the overall phosphorylation level of S6K1 was upregulated in cells with reintroduced catalytically inactive OTUD3C76A (Figures 3E, F). This suggests that OTUD3C76A, lacking deubiquitinase activity, cannot reverse the upregulation of the mTORC1 signaling pathway caused by the knockout of endogenous OTUD3.

In this section of the study, through a series of experiments involving amino acid starvation and stimulation in HeLa cells, we demonstrated that OTUD3 can downregulate the mTORC1 signaling pathway, and this downregulation is dependent on the enzymatic deubiquitinase activity of OTUD3.

### 3.4 OTUD3 downregulated mTORC1 not only relies on the PTEN/Akt route but also mainly on the KPTN via the KICSTOR pathway

PTEN is a significant deubiquitinating substrate of OTUD3, the protein level of PTEN is regulated by the deubiquitination function of OTUD3. OTUD3 stabilizes PTEN protein levels, thereby regulating the Akt signaling pathway (Yuan et al., 2015). While OTUD3 protein can indirectly downregulate the mTORC1 signaling pathway, does its impact rely on stabilizing PTEN protein levels, or is it unrelated to the deubiquitination process of KPTN protein discussed earlier?

**FIGURE 4:**
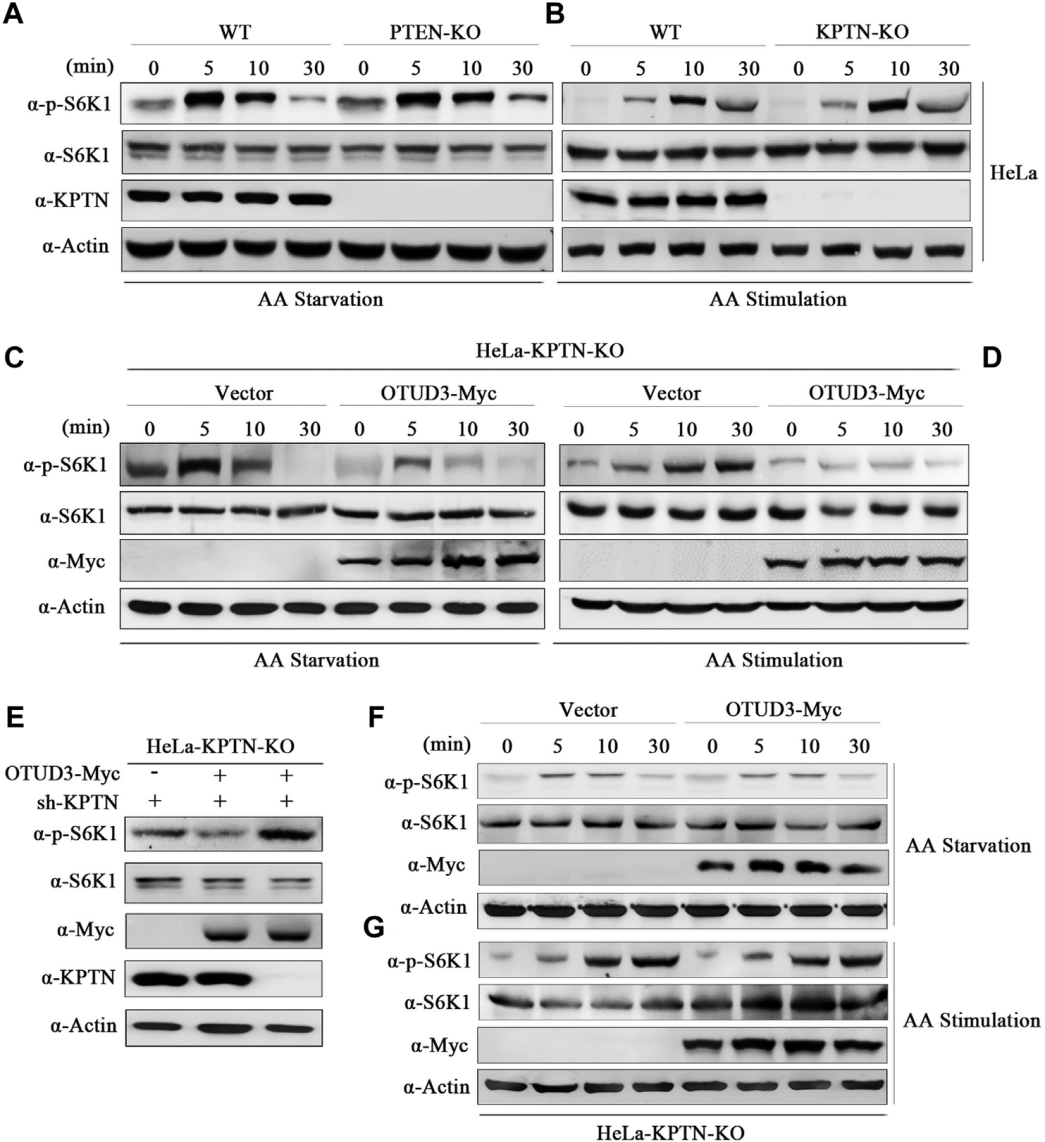
OTUD3 downregulated mTORC1 not only relies on the PTEN/Akt route but also mainly on the KPTN via the KICSTOR pathway. **(A)** Wild-type HeLa cells or HeLa cells with PTEN knockout were subjected to a time-gradient amino acid starvation treatment using amino acid-free basal culture medium. Changes in S6K1 phosphorylation were monitored. **(B)** Wild-type HeLa cells or HeLa cells with PTEN knockout were subjected to amino acid starvation for 1 h using amino acid-free basal culture medium, followed by direct addition of amino acid solution for a specified duration. Changes in S6K1 phosphorylation were examined. **(C)** HeLa cells with PTEN knockout and exogenous expression of OTUD3-Myc or empty vector were subjected to a time-gradient amino acid starvation treatment using amino acid-free basal culture medium. Changes in S6K1 phosphorylation were monitored. **(D)** HeLa cells with PTEN knockout and exogenous expression of OTUD3-Myc or empty vector were subjected to amino acid starvation for 1 h using amino acid-free basal culture medium, followed by direct addition of amino acid solution for a specified duration. Changes in S6K1 phosphorylation were examined. **(E)** Within PTEN knockout HeLa cells, exogenous expression of OTUD3-Myc and transfection with shRNA targeting KPTN were performed to monitor changes in S6K1 phosphorylation. **(F)** HeLa cells with KPTN knockout and exogenous expression of OTUD3-Myc or empty vector were subjected to a time-gradient amino acid starvation treatment using amino acid-free basal culture medium. Changes in S6K1 phosphorylation were examined. **(G)** HeLa cells with KPTN knockout and exogenous expression of OTUD3-Myc or empty vector were subjected to amino acid starvation for 1 h using amino acid-free basal culture medium, followed by direct addition of amino acid solution for a specified duration. Changes in S6K1 phosphorylation were monitored. “AA” stands for amino acid.

To address these questions, we conducted gene knockout experiments targeting the PTEN gene in HeLa cells using the Crispr/Cas9 system. We selected wild-type HeLa cells and PTEN-knockout HeLa cells (PTEN-KO), subjecting them to time-gradient amino acid starvation or amino acid starvation followed by stimulation. Finally, cell samples were collected and subjected to immunoblotting using corresponding endogenous antibodies. Results indicated that, compared to wild-type HeLa cells, the overall phosphorylation level of S6K1 was elevated in PTEN-KO HeLa cells, confirming that PTEN indeed downregulates the mTORC1 signaling pathway (Figures 4A, B). In subsequent experiments, we exogenously expressed OTUD3-Myc in PTEN-knockout HeLa cells and subjected these cells to time-gradient amino acid starvation or amino acid starvation followed by stimulation. The results showed that, in cells expressing exogenous OTUD3, the overall phosphorylation level of S6K1 was downregulated in comparison to PTEN-knockout HeLa cells (Figures 4C, D). This finding indicates that the upregulation of the mTORC1 signaling pathway caused by PTEN-KO cells can be suppressed by exogenous expression of OTUD3-Myc. This result further supports the notion that besides influencing the mTORC1 signaling pathway through stabilizing PTEN protein levels, OTUD3 possesses alternative pathways independent of PTEN’s presence to modulate mTORC1 signaling.

OTUD3 appears to downregulate the mTORC1 signaling pathway through a PTEN-independent route, does this pathway possibly involve the modulation of KPTN ubiquitination? To test this hypothesis, we transfected KPTN shRNA into PTEN-KO HeLa cells exogenously expressing OTUD3-Myc, observing whether OTUD3 could still inhibit the mTORC1 signaling pathway in the absence of both PTEN and KPTN. The results showed that the ability of OTUD3 to inhibit the mTORC1 signaling pathway was significantly weakened under conditions of concurrent PTEN and KPTN depletion (Figure 4E). This suggests that OTUD3’s ability to inhibit the mTORC1 signaling pathway relies not only partially on the PTEN/Akt route but also significantly on the KPTN (via KICSTOR) pathway.

To further validate this hypothesis, we conducted gene knockout experiments targeting the KPTN gene in HeLa cells using the Crispr/Cas9 system. In KPTN-knockout HeLa cells, we exogenously transfected OTUD3-Myc and empty vector, subjecting these cells to time-gradient amino acid starvation or amino acid starvation followed by stimulation. The results indicated that in KPTN-knockout HeLa cells, exogenously expressed OTUD3-Myc did not exhibit the same ability to significantly reduce the overall phosphorylation level of S6K1 as shown previously, and the levels were comparable to those of KPTN-knockout cells transfected with the empty vector (Figures 4F, G). These findings partially confirm the essential role of the KPTN protein in OTUD3’s ability to downregulate the mTORC1 signaling pathway in cells.

### 3.5 OTUD3 mediated the lysosomal localization of GATOR1 and this process requires the participation of KPTN

To consider whether OTUD3’s deubiquitination of KPTN might affect the ability of the KICSTOR protein complex to recruit GATOR1 to the lysosomal surface, we designed the following experiment. We exogenously expressed NPRL2-Flag and LAMP2-mcherry as markers for GATOR1 and lysosomes, respectively, in WT, OTUD3-KO and KPTN-KO HeLa cells. Additionally, we transfected OTUD3-Myc as an experimental group in HeLa cells with endogenous OTUD3 knocked out or HeLa cells with endogenous KPTN knocked out.

**FIGURE 5:**
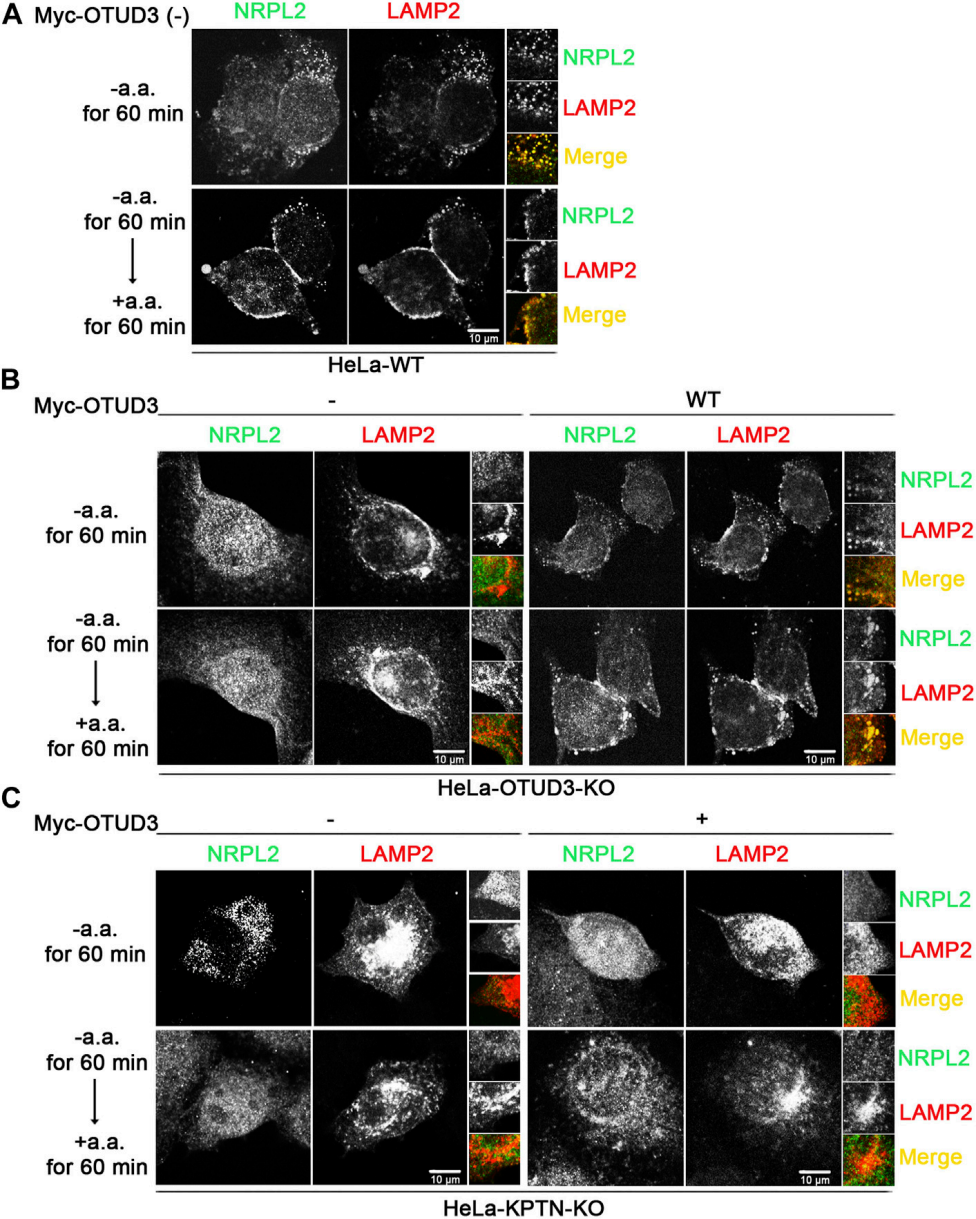
OTUD3 mediated the lysosomal localization of GATOR1 and this process requires the participation of KPTN. **(A)** Co-localization of NPRL2 and LAMP2 in wild-type HeLa cells. NPRL2 exhibits amino acid-insensitive lysosomal localization. **(B)** Co-localization of NPRL2 and LAMP2 in HeLa cells with OTUD3 knockout and in cells with exogenous OTUD3 protein complementation. In the absence of OTUD3 protein, NPRL2 loses its lysosomal localization, while complementation with exogenous OTUD3 protein restores NPRL2’s lysosomal localization. **(C)** Co-localization of NPRL2 and LAMP2 in HeLa cells with KPTN knockout and in cells with exogenous OTUD3 protein complementation. In the absence of KPTN protein, NPRL2 loses its lysosomal localization. Interestingly, complementation with exogenous OTUD3 protein cannot restore NPRL2’s lysosomal localization. Scale bar, 10 μm. The inset on the right is magnified 3 times.

Subsequently, we subjected these cells to amino acid starvation and amino acid starvation followed by stimulation, obtained samples, and performed immunostaining. We then observed the localization of NPRL2 and LAMP2 under a fluorescence microscope to investigate the lysosomal localization of GATOR1 under different condition. From the immunofluorescence images, we found that in wild-type HeLa cells (HeLa-WT) containing OTUD3 protein, NPRL2 protein co-localized with lysosomes in an amino acid-insensitive manner (Figure 5A). However, in OTUD3-knockout HeLa cells (HeLa-OTUD3-KO), NPRL2 protein was evenly distributed throughout the cytoplasm and no longer localized to lysosomes. Interestingly, when we reintroduced exogenous OTUD3 protein into HeLa-OTUD3-KO cells (HeLa-KPTN-KO), NPRL2 was once again recruited to lysosomes (Figure 5B). In contrast, in HeLa-KPTN-KO cells, irrespective of whether exogenous OTUD3 protein was overexpressed, NPRL2 was evenly distributed in the cytoplasm (Figure 5C).

These results suggest that GATOR1 localizes to lysosomes in normal cells. In the absence of OTUD3 protein, GATOR1’s lysosomal localization is hindered, resulting in a tendency to distribute evenly throughout the cytoplasm. However, the reintroduction of exogenous OTUD3 protein can restore GATOR1’s lysosomal localization. Notably, in the absence of KPTN protein, even with exogenous OTUD3 protein reintroduced, GATOR1 cannot regain lysosomal localization. This implies that although the OTUD3 protein can influence GATOR1’s lysosomal localization, this process requires the participation of the KPTN protein. A plausible consequence of this scenario is that when cells lack OTUD3 protein, it could lead to impaired lysosomal localization of GATOR1, thereby reducing GATOR1’s inhibitory function and resulting in an elevation of the mTORC1 signaling pathway. This, in turn, would enable cells to activate the mTORC1 pathway even under low amino acid conditions, enhancing their tolerance to amino acid starvation and ultimately promoting the growth and proliferation of tumor cells.

### 3.6 OTUD3 suppressed proliferation and growth of HeLa by downregulating the mTORC1

**FIGURE 6:**
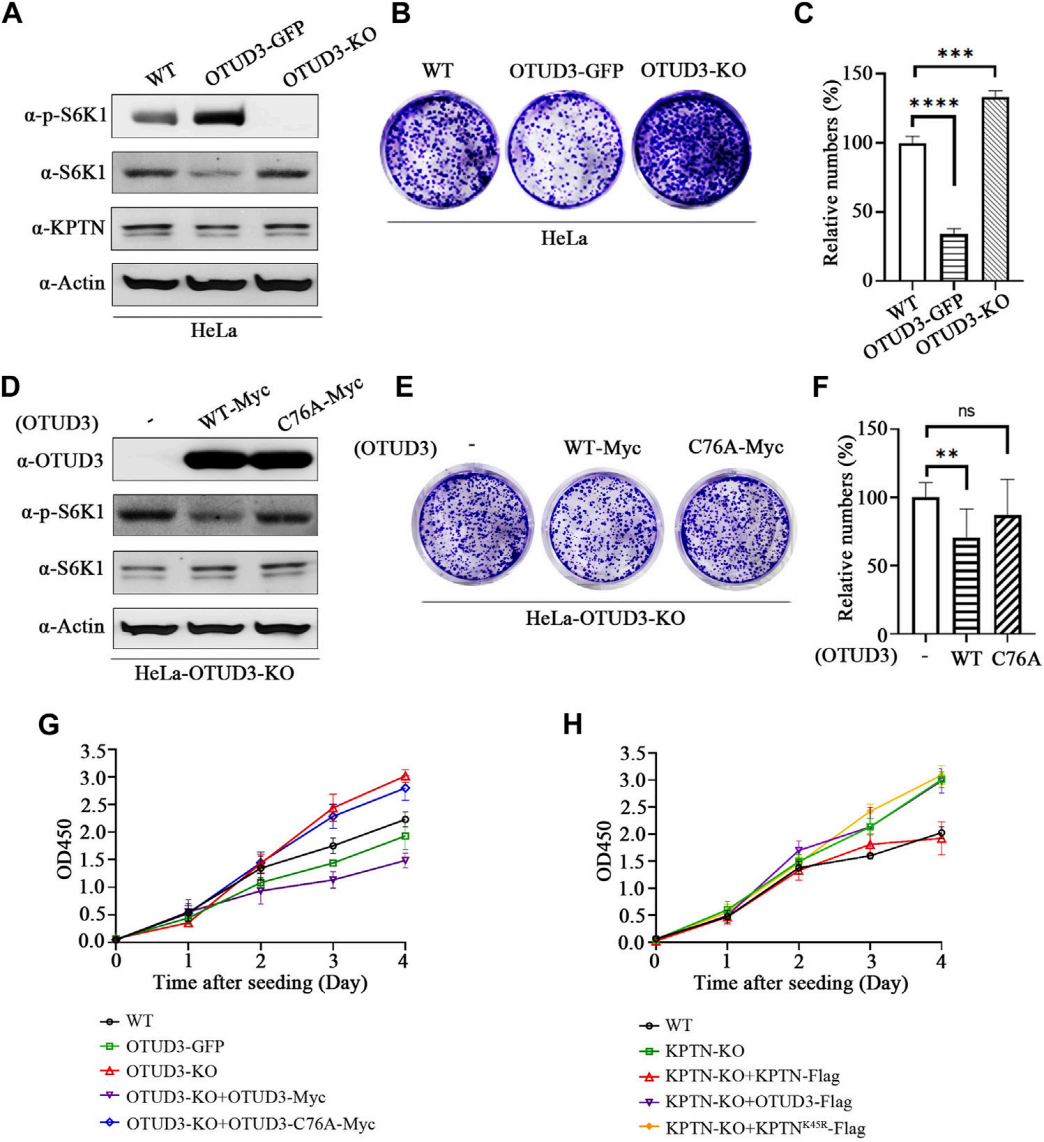
OTUD3 Suppressed proliferation and growth of HeLa by downregulating the mTORC1. **(A)** Detection of indicated protein levels in wild-type HeLa cells, HeLa cells stably overexpressing OTUD3, or HeLa cells with OTUD3 knockout. **(B)** Example images of crystal violet staining experiments in wild-type HeLa cells, HeLa cells stably overexpressing OTUD3, or HeLa cells with OTUD3 knockout. **(C)** Relative cell counts based on crystal violet staining in wild-type HeLa cells, HeLa cells stably overexpressing OTUD3, or HeLa cells with OTUD3 knockout. The results are shown as relative percentages ±SD, with the mean count in HeLa-WT cells set to 100%. *n* = 3 independent experiments. ***, *p* < 0.001; ****, *p* < 0.0001, Student’s t-test. **(D)** Detection of indicated protein levels in HeLa-OTUD3-KO cells overexpressing wild-type (WT) or catalytically inactive OTUD3C76A. **(E)** Example images of crystal violet staining experiments in HeLa-OTUD3-KO cells overexpressing wild-type (WT) or catalytically inactive OTUD3C76A. **(F)** Relative cell counts based on crystal violet staining in wild-type, HeLa-OTUD3-KO cells overexpressing wild-type (WT) or catalytically inactive OTUD3C76A. The results are shown as relative percentages ±SD, with the mean count in HeLa-OTUD3-KO cells set to 100%. *n* = 3 independent experiments. **, *p* < 0.01, Student’s t-test. **(G)** Proliferation curves of five different HeLa cell types using the CCK-8 reagent: ① Wild-type HeLa; ② HeLa with stable endogenous expression of OTUD3-GFP; ③ HeLa with OTUD3 knockout; ④ OTUD3 knockout HeLa with exogenous expression of OTUD3-Myc; ⑤ OTUD3 knockout HeLa with exogenous expression of OTUD3C76A-Myc. The results are shown as mean OD450 values ±SD, *n* = 6 independent experiments. **(H)** Proliferation curves of five different HeLa cell types using the CCK-8 reagent: ① Wild-type HeLa; ② KPTN knockout HeLa; ③ KPTN knockout HeLa with exogenous expression of KPTN-Flag; ④ KPTN knockout HeLa with exogenous expression of OTUD3-Flag; ⑤ OTUD3 knockout HeLa with exogenous expression of KPTNK49R-Flag. The results are shown as mean OD450 values ±SD, *n* = 6 independent experiments.

We know that downregulation of mTORC1 usually implies inhibition of tumor cell growth. The following experiments were conducted to investigate the impact of this process on tumor cell growth. A stable overexpressing OTUD3 (OTUD3-GFP) HeLa cell line was generated (Figure 6A), then the growth of wild-type HeLa cells (WT), OTUD3-GFP HeLa cells, and OTUD3 knockout (OTUD3-KO) HeLa cells were compared using crystal violet staining. The results indicated that stable expression of OTUD3 inhibited HeLa cell growth, while OTUD3 knockout promoted HeLa cell growth (Figures 6B, C). Furthermore, we performed crystal violet staining experiments in HeLa-OTUD3-KO cells with exogenous expression of either wild-type OTUD3 (WT-Myc) or catalytically inactive OTUD3 (C76A-Myc) (Figure 6D). The results showed that reintroducing wild-type OTUD3 into OTUD3 knockout HeLa cells significantly inhibited their growth (group WT-Myc), while reintroducing catalytically inactive OTUD3 had no significant impact on growth (group C76A-Myc) (Figures 6E, F). These experiments collectively confirmed that OTUD3 can suppress the growth and proliferation of tumor cells.

Moreover, to corroborate these findings, we conducted CCK8 cell proliferation assays on HeLa-WT, OTUD3-GFP, OTUD3-KO, OTUD3-KO cells with exogenous expression of wild-type OTUD3 (OTUD3-KO+OTUD3-Myc), and OTUD3-KO cells with exogenous expression of catalytically inactive OTUD3 (OTUD3-KO+OTUD3-C76A-Myc). The results revealed that stable expression of OTUD3 inhibited HeLa cell proliferation, OTUD3 knockout promoted proliferation, and reintroduction of wild-type OTUD3 into OTUD3-KO cells restored the enhanced proliferation ability. However, reintroduction of catalytically inactive OTUD3 had no such effect (Figure 6G). It is noteworthy that OTUD3-KO cells overexpressing wild-type OTUD3 exhibited lower proliferation rates compared to cells with stable overexpression of OTUD3-GFP. This might be due to the transient overexpression of exogenous OTUD3-Myc leading to significantly higher protein levels than the stable overexpression of endogenous OTUD3, resulting in higher suppression of the mTORC1 signaling pathway and influencing HeLa cell proliferation rates. This observation is consistent with our earlier results.

Meanwhile, we conducted CCK8 cell proliferation assays on HeLa-WT, KPTN-KO, KPTN-KO cells with exogenous expression of wild-type KPTN (KPTN-KO+KPTN-Flag), KPTN-KO cells with exogenous expression of wild-type OTUD3 (KPTN-KO+OTUD3-Flag), and KPTN-KO cells with exogenous expression of KPTN that cannot be ubiquitinated (KPTN-KO+KPTNK45R-Flag). The results indicated that KPTN knockout promoted HeLa cell proliferation. Overexpressing wild-type KPTN in KPTN-KO cells reduced the enhanced proliferation ability, while overexpressing KPTN that cannot be ubiquitinated and overexpressing wild-type OTUD3 both significantly increased proliferation rates compared to wild-type HeLa cells, equivalent to KPTN-KO cells (Figure 6H).

Collectively, these data suggest OTUD3’s function in reducing tumor cell proliferation requires the presence of KPTN protein, and the ability of KPTN to inhibit tumor cell proliferation relies on the presence of its ubiquitination site.

### 3.7 OTUD3 suppressed proliferation and growth of HeLa by downregulating mTOR signaling mediated cell metabolism

**FIGURE 7:**
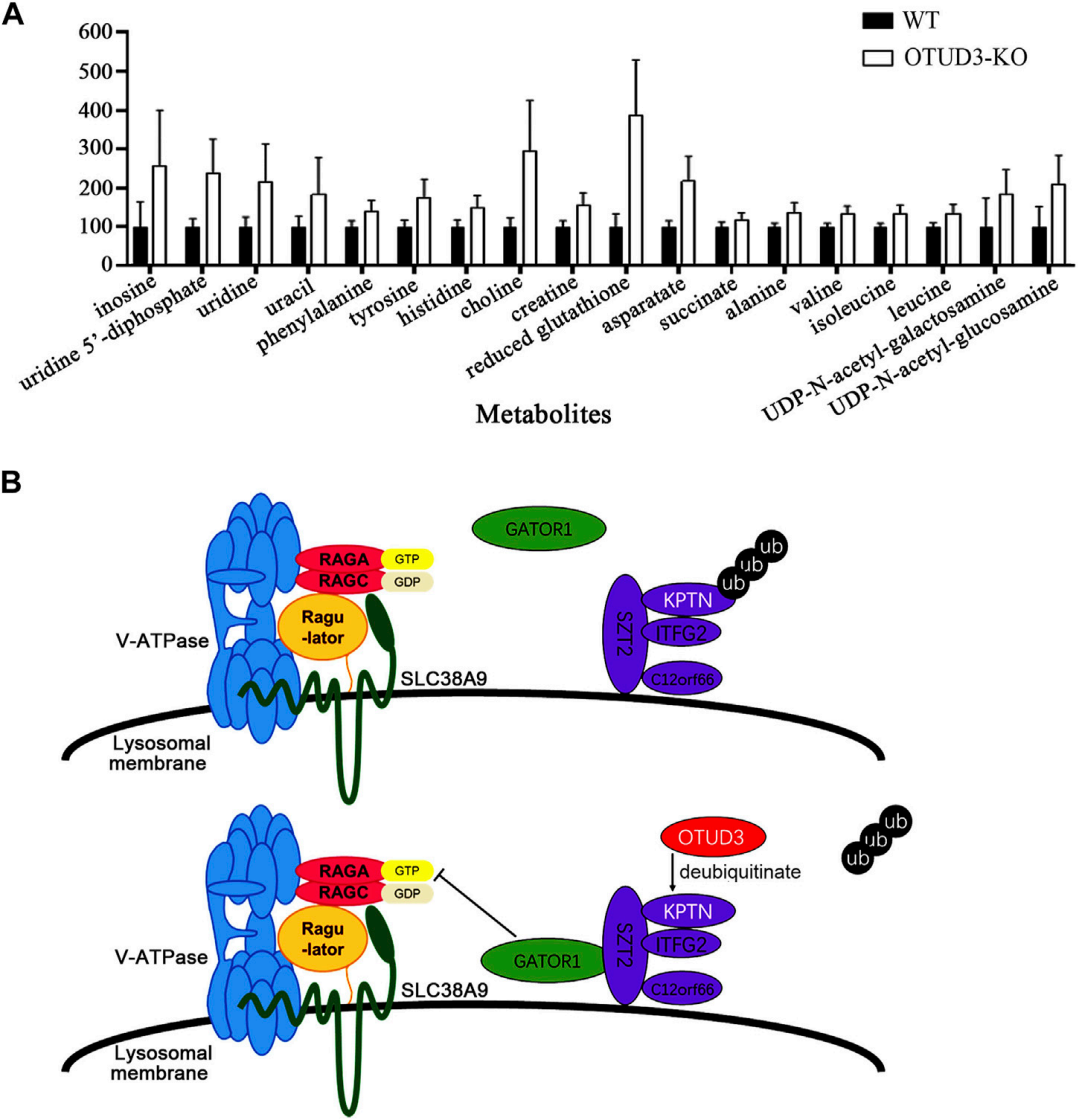
OTUD3 Suppressed proliferation and growth of HeLa by downregulating mTOR signaling mediated cell metabolism. **(A)** Metabolites significantly different between OTUD3 knockout HeLa cells and wild-type HeLa cells. The results are shown as relative percentages ±SD, with the mean in WT-HeLa set to 100%, based on 12 independent experiments. Student’s t-test was performed. UDP-N-acetyl-galactosamine group, uracil group, succinate group have *p* < 0.01; other metabolite groups have *p* < 0.001. **(B)** Schematic representation of OTUD3’s role in regulating the mTORC1 signaling pathway through deubiquitinating KPTN. OTUD3 interacts with KPTN and utilizes its deubiquitinase activity to remove ubiquitin modifications from KPTN, allowing the KICSTOR protein complex to recruit GATOR1 from the cytoplasm, enabling GATOR1 to inhibit the mTOR protein complex. Loss or dysfunction of OTUD3 in cells leads to the KICSTOR complex losing the ability to recruit GATOR1, resulting in the activation of the mTOR protein complex.

To further explore the metabolic differences between OTUD3-KO and wild-type HeLa cells, we performed nuclear magnetic resonance (NMR) spectroscopy on both cell types, analyzing a total of 30 metabolites. Among them, 18 metabolites exhibited significant differences in content between OTUD3-KO and WT HeLa cells (Figure 7A).

Notably, eight of these metabolites are amino acids, including phenylalanine, tyrosine, histidine, aspartic acid, alanine, valine, isoleucine, and leucine. The increase in levels of these metabolites, especially essential amino acids, suggests increased amino acid uptake for biosynthesis. The elevated level of succinate indicates a shift towards glycolytic metabolism and the accumulation of intermediates, aligning with the well-known “Warburg effect” (Palsson–McDermott and O'neill, 2013). The high levels of reduced glutathione are favorable for maintaining oxidative stress levels, providing protection for rapidly growing tumor cells (Pani et al., 2010). The increased levels of UDP, uridine, and uracil suggest enhanced RNA synthesis (Dobolyi et al., 2011), likely linked to increased mRNA transcription levels due to activation of the mTOR signaling pathway and heightened protein synthesis activities. The elevated levels of UDP-N-acetylglucosamine and UDP-N-acetylgalactosamine are crucial substrates in protein modification processes within tumor cells, influencing various physiological processes including transformation and metastasis (Itkonen et al., 2015). Their increased levels support the rapid growth and proliferation needs of tumor cells.

In this section of the study, we validated, at the cellular level through crystal violet staining and CCK8 cell proliferation assays, that OTUD3 has the capacity to inhibit tumor cell growth and proliferation by downregulating the mTOR signaling pathway. Additionally, we used NMR to examine the content levels of various metabolites between OTUD3-KO and wild-type HeLa cells, identifying several metabolites supporting the rapid growth and proliferation of tumor cells that were significantly elevated in OTUD3-KO cells. These results collectively support OTUD3’s ability to suppress tumor cell growth and proliferation through the downregulation of the mTOR signaling pathway.

## 4 Discussion

Ubiquitination modification has long been an area of keen interest for researchers, especially in the field of protein regulation (Wang et al., 2022; Zhang et al., 2023b). The discovery that ubiquitination targets proteins for proteasomal degradation earned the Nobel Prize in Chemistry in 2004. The elucidation of ubiquitination and its functions has enriched our understanding of the complete journey of a protein’s life cycle - from translation initiation, localization to specific cellular compartments for function, to eventual degradation within the proteasome (Qi et al., 2023). This comprehensive view allows us to witness the journey from protein “infancy” to mature functionality and finally to its “senescent” state. Like fallen leaves nourishing the Earth, ubiquitinated proteins are transformed into amino acids, serving as building blocks for other proteins and biomolecules, rejuvenating the cellular landscape. Alternatively, they can be oxidized into urea and carbon dioxide, serving as energy sources for life processes (Miflin and Lea, 1977), resembling the fuel for the fire of life. They might also serve as intermediates in various metabolic pathways, like glycine serving as the precursor for porphyrins in heme (Shemin and Eittenberg, 1946), orchestrating diverse life activities. The selective degradation of proteins within the proteasome is intricately regulated in terms of timing and spatial control, with deubiquitinases (DUBs) being integral components in this precision (Yan et al., 2020). They play pivotal roles in cellular homeostasis and developmental processes, exerting significant influence on various aspects of cell life. While our understanding of deubiquitination is nascent compared to ubiquitination, it is recognized as a vital component of the ubiquitin system, particularly when associated with the cell cycle (Song and Rape, 2008).

The journey of exploring deubiquitination has just commenced, especially for the OTUD deubiquitinase superfamily. Through bioinformatics analyses of genomic databases, researchers have identified the evolutionary conservation of the OTUD deubiquitinase superfamily and detected mutations in its members linked to genetic diseases, immune inflammation, and tumor development. In breast cancer, OTUD3 stabilizes PTEN, inhibiting the PI3K/p-AKT/mTOR pathway and suppressing tumor growth. Regulation by miR-520h (Geng et al., 2020) and miR-32 (Jin et al., 2019) influences cell behavior, affecting proliferation and drug resistance (Zhang et al., 2020). OTUD3, regulated by CHIP (Zhang et al., 2020), impacts lung cancer invasion and metastasis (Zhang et al., 2023c). Inhibition by OTUDin3 shows promise in non-small cell lung cancer (Zhang et al., 2023c). OTUD3 also influences hepatocellular carcinoma metastasis by mediating ACTN4 deubiquitination (Xie et al., 2021). In lung cancer, OTUD3 interacts with GRP78, inhibiting cell growth and migration (Du et al., 2019). OTUD3 shows varied effects in gliomas and squamous cell carcinomas. Lower expression in gliomas suggests involvement in gliomagenesis, while OTUD3 locus deletion may contribute to squamous cell carcinoma (Liu et al., 2020; Gatti et al., 2020). Clinical evidence is needed to clarify its role and potential for targeted therapy. Through the analysis of the GEPIA2 database and the expression level differences of OTUD3 in various tumors and corresponding control tissues from The Cancer Genome Atlas (TCGA), we observed a significant downregulation of OTUD3 in multiple tumors such as Ovarian Serous Cystadenocarcinoma (OV), Testicular Germ Cell Tumors (TGCT), Uterine Corpus Endometrial Carcinoma (UCEC), and Uterine Carcinosarcoma (UCS). Simultaneously, we evaluated the impact of OTUD3 expression differences on the survival rates in patients with these four types of tumors. We found that among patients with these tumors, the overall survival rate of the high OTUD3 expression group was significantly better than that of the low OTUD3 expression group. Additionally, in the survival analysis, patients with high OTUD3 expression demonstrated a better Relapse-Free Survival (RFS) rate compared to those with low expression. This series of results suggests that OTUD3 may play a role as a tumor suppressor gene in various cancers. Nevertheless, our understanding of the roles and regulation of the OTUD deubiquitinase superfamily in various cellular metabolic processes from a cellular, metabolic network, and molecular mechanism perspective remains limited.

In our study, we uncovered a previously unreported substrate of the deubiquitinase OTUD3 - KPTN. As a member of the KICSTOR protein complex, KPTN’s presence is crucial for the regulation of the mTORC1 pathway in cells. KICSTOR is thought to negatively regulate the Rag-GTPase signal sent to mTORC1 by recruiting GATOR1 to lysosomes. Deficiency of KICSTOR members in cells leads to effects similar to the loss of GATOR1, causing abnormal activation of the mTORC1 signaling pathway and enhanced tolerance to low amino acid environments. We found that OTUD3 significantly deubiquitinates KPTN, effectively removing polyubiquitin chains on the lysine 49 residue. However, OTUD3’s deubiquitination of KPTN does not affect its protein levels. In other words, KPTN ubiquitination does not lead to its degradation pathway. This prompted us to further investigate OTUD3’s role in KPTN deubiquitination and its effect on the mTORC1 pathway. We started by observing a phenomenon: OTUD3 can reduce the levels of the mTORC1 signaling pathway. It accelerates the decrease in S6K1 phosphorylation levels when amino acids are deficient and delays the increase in S6K1 phosphorylation levels when amino acids are stimulated. We confirmed that this modulation is due to the enzymatic activity of OTUD3’s deubiquitinase, rather than other intrinsic properties. Furthermore, OTUD3’s downregulation of the mTORC1 pathway requires the presence of its substrate, KPTN. When KPTN is knocked out, OTUD3 no longer significantly affects the mTORC1 pathway. Through immunofluorescence, we also observed that OTUD3 affects the intracellular distribution of NPRL2, a marker of the GATOR1 complex. GATOR1, composed of DEPDC5, NPRL2, and NPRL3, negatively regulates mTORC1 as it responds to amino acid deficiency, inhibiting the mTORC1 signaling pathway by inactivating Rag-GTPases via its GAP activity. GATOR1’s inhibitory function is localized to lysosomes (Wolfson et al., 2017). In cells lacking OTUD3, we found that NRPL2 loses its lysosomal localization, and this is restored when exogenous OTUD3 is reintroduced. Notably, OTUD3’s effect on NPRL2’s lysosomal localization requires KPTN’s participation. In KPTN-knockout cells, OTUD3’s presence fails to ensure NPRL2’s lysosomal localization.

The potential consequence of OTUD3’s downregulation of the mTORC1 pathway could be the suppression of tumor cell growth and proliferation. Cellular experiments support this hypothesis, as OTUD3-knockout cells exhibited accelerated growth and proliferation, while cells with stable OTUD3 overexpression displayed delayed growth and proliferation. Our metabolic analysis of OTUD3-knockout and wild-type cells also demonstrated significant increases in various metabolite levels in the former, including succinate, reduced glutathione, essential amino acids, nucleotide-related metabolites, etc. These metabolites contribute to aerobic glycolysis (the Warburg effect), oxidative stress response, protein synthesis, transcription, and other processes that support tumor cell growth and proliferation. The rise in succinate suggests an upregulation of the TCA cycle within cells. Aerobic glycolysis allows tumor cells to maintain high levels of ATP production even in hypoxic conditions, and the accumulation of carbon products in the TCA cycle provides ample precursors for biosynthesis. The mTORC1 pathway enhances the expression of genes involved in glucose uptake and glycolysis through its transcription factor hypoxia-inducible factor 1-alpha (HIF1α) (Hudson et al., 2002). The pyrimidine nucleotide biosynthesis enzyme CAD is a key component in *de novo* synthesis. Research suggests that the mTOR complex-associated protein mLST8 interacts with CAD through multiple binding regions, mediating mTOR’s regulation of CAD’s enzymatic activity, illustrating the role of the mTOR signaling pathway in pyrimidine *de novo* synthesis (Prives and Quastel, 1969). OTUD3’s knockout leads to abnormal mTORC1 pathway activation, resulting in Warburg effect induction and increased synthesis-related metabolism.

While our study elucidates how OTUD3 regulates the mTORC1 pathway by deubiquitinating KPTN and influencing the recruitment of the KICSTOR complex to GATOR1, many aspects remain to be fully explored. For instance, what is the ubiquitination enzyme responsible for KPTN ubiquitination? Does it work in conjunction with OTUD3 to regulate the mTORC1 pathway? What molecular mechanisms underlie KPTN’s ubiquitination affecting the recruitment of the KICSTOR complex to GATOR1 at lysosomes? Does it involve direct modulation of the KICSTOR-GATOR1 interaction or other protein-mediated processes? Other components of the KICSTOR complex may also be ubiquitinated, but OTUD3’s deubiquitination does not affect them. Do they possess their own specific ubiquitination/deubiquitination proteins, yet to be discovered? Do they, like OTUD3, play roles in regulating the mTORC1 signaling pathway? These questions provide directions for our future research. We should focus on the yet-to-be-fully-understood regulatory mechanisms brought about by ubiquitination modifications in the mTORC1 signaling pathway.

## 5 Conclusion

In summary, our experiments shed light on OTUD3’s intrinsic role in inhibiting tumor initiation and development, proposing a novel mechanism involving KPTN in mTORC1 pathway regulation. This offers a fresh perspective on the occurrence and progression of tumor diseases caused by relevant genes and provides inspiration for drug screening and cancer treatment. It presents a potential roadmap for future therapies targeting relevant tumors.

## Data Availability

The original contributions presented in the study are included in the article/supplementary materials, further inquiries can be directed to the corresponding authors.
